# Flight-Associated Transmission of Severe Acute Respiratory Syndrome Coronavirus 2 Corroborated by Whole-Genome Sequencing

**DOI:** 10.3201/eid2612.203910

**Published:** 2020-12

**Authors:** Hollie Speake, Anastasia Phillips, Tracie Chong, Chisha Sikazwe, Avram Levy, Jurissa Lang, Benjamin Scalley, David J. Speers, David W. Smith, Paul Effler, Suzanne P. McEvoy

**Affiliations:** University of Notre Dame, Fremantle, Western Australia, Australia (H. Speake);; Metropolitan Communicable Disease Control, Perth, Western Australia, Australia (A. Phillips, T. Chong, B. Scalley, S.P. McEvoy);; University of Western Australia, Perth (C. Sikazwe, A. Levy, D.J. Speers, P. Effler);; PathWest Laboratory Medicine Western Australia, Nedlands, Western Australia, Australia (C. Sikazwe, A. Levy, D.J. Speers, D.W. Smith, J. Lang);; Public Health Emergency Operations Centre, Perth (P. Effler)

**Keywords:** 2019 novel coronavirus disease, coronavirus disease, COVID-19, severe acute respiratory syndrome coronavirus 2, SARS-CoV-2, viruses, respiratory infections, zoonoses, aircraft, in-flight, flight-associated, transmission, airport, whole-genome sequencing, cruise, outbreak

## Abstract

To investigate potential transmission of severe acute respiratory syndrome coronavirus 2 (SARS-CoV-2) during a domestic flight within Australia, we performed epidemiologic analyses with whole-genome sequencing. Eleven passengers with PCR-confirmed SARS-CoV-2 infection and symptom onset within 48 hours of the flight were considered infectious during travel; 9 had recently disembarked from a cruise ship with a retrospectively identified SARS-CoV-2 outbreak. The virus strain of those on the cruise and the flight was linked (A2-RP) and had not been previously identified in Australia. For 11 passengers, none of whom had traveled on the cruise ship, PCR-confirmed SARS-CoV-2 illness developed between 48 hours and 14 days after the flight. Eight cases were considered flight associated with the distinct SARS-CoV-2 A2-RP strain; the remaining 3 cases (1 with A2-RP) were possibly flight associated. All 11 passengers had been in the same cabin with symptomatic persons who had culture-positive A2-RP virus strain. This investigation provides evidence of flight-associated SARS-CoV-2 transmission.

On March 21, 2020, the Western Australia Department of Health was notified that 6 passengers aboard a flight from Sydney, New South Wales, to Perth, Western Australia, Australia, on March 19 had tested positive for severe acute respiratory syndrome coronavirus 2 (SARS-CoV-2) by PCR. All 6 passengers had disembarked from cruise ships that had recently docked in Sydney. In the subsequent 2 weeks, several other cases of SARS-CoV-2 infection were identified among passengers on that flight.

Although the role of cruise ships in SARS-CoV-2 transmission is well documented (*1*), information regarding potential flight-associated transmission of SARS-CoV-2 (*2*,*3*) is limited. We investigated SARS-CoV-2 transmission associated with a 5-hour domestic flight by analyzing epidemiologic and whole-genome sequencing (WGS) data. Ethics approval was not required for this investigation, conducted as part of the public health response to the SARS-CoV-2 outbreak under the Western Australia Public Health Act 2016.

## Methods

### Public Health Response to Coronavirus Disease in Australia

In Australia, coronavirus disease (COVID-19) is an urgently notifiable disease (*4*); laboratory-confirmed cases and close contacts are investigated and managed according to national guidelines produced by the Communicable Disease Network of Australia (*4*). Details for flights with SARS-CoV-2 infectious persons on board are published at https://www.healthywa.wa.gov.au/coronavirus. Airlines are responsible for the management of crew and are notified of potential in-flight exposure by the National Incident Room (https://www.health.gov.au/initiatives-and-programs/national-incident-room).

### Initial Public Health Investigation of the Flight

On the afternoon of March 19, 2020, a domestic flight within Australia departed Sydney and landed 5 hours later in Perth. The aircraft, an Airbus A330-200, had 28 business and 213 economy class passengers on board. Passengers were persons transiting to Perth through the Sydney International Airport after arriving from overseas and domestic travelers, some of whom had disembarked from 1 of 3 cruise ships that had recently docked in Sydney Harbour (Ovation of the Seas on March 18; Ruby Princess and Sun Princess on March 19).

After the initial 6 persons with COVID-19 were identified among passengers on the flight, all close contacts were informed of their potential exposure to SARS-CoV-2 and directed to quarantine themselves for 14 days. During this investigation, PCR testing for SARS-CoV-2 was limited to persons experiencing symptoms. By April 1, SARS-CoV-2 infection was confirmed for >20 passengers on the flight, and attempts were made to notify all remaining passengers of their potential exposure. The number of infections linked to the flight and the timing of symptom onset among persons with a later diagnosis suggested that flight-associated transmission may have occurred.

### Laboratory Methods

For patients with suspected SARS-CoV-2 infection, a throat swab and bilateral nasopharyngeal or deep nasal specimens were collected on flocked nasopharyngeal swabs. The swabs were placed in viral, universal, or Liquid Amies (https://www.copanusa.com) transport medium, then transported and stored at 4°–8°C. Testing was conducted at PathWest Laboratory Medicine WA by use of either the Roche cobas SARS-CoV-2 test (Roche Diagnostics, https://www.roche.com) or combined in-house assays directed at envelope and spike protein gene targets (Table 1) (*5*). The in-house assays were approved for diagnostic use by the National Association of Testing Authorities according to the provisions of the National Pathology Accreditation Advisory Council (*6*).

**Table 1:** Primers and probe sequences used for in-house diagnostic assays used to test persons with suspected SARS-CoV-2 infection after flight from Sydney to Perth, Australia, March 19, 2020

Oligonucleotide	Sequence, 5¢®3¢	Concentration, μM
BetaCoV_Wuhan_F22595 (forward)	TGAAGTTTTTAACGCCACCAGAT	0.7
BetaCoV_Wuhan_R22662 (reverse)	CACAGTTGCTGATTCTCTTCCTGTT	0.9
BetaCoV_Wuhan_P22619-HEX (hydrolysis probe)	(HEX)C[+A][+A]GCAT[+A][+A]A[+C]AGATGCA(BHQ1)	0.3
MS2-105F (forward)	GTCGACAATGGCGGAACTG	0.2
MS2-170R (reverse)	TTCAGCGACCCCGTTAGC	0.5
MS2-127-VIC (hydrolysis probe)	(VIC)ACGTGACTGTCGCCCCAAGCAACTT(QSY)	0.2

When a sample was available for genomic testing, it underwent tiled amplicon PCR at PathWest to generate 14 overlapping amplicons representing the whole genome (Appendix Table), which were then sequenced on an Illumina MiSeq sequencer (Illumina, https://www.illumina.com). If multiple samples were available from 1 patient, we used samples with the highest viral loads (i.e., lowest cycle threshold values). Processed reads were mapped to the SARS-CoV-2 reference genome (GenBank accession no. MN908947). Primer-clipped alignment files were imported into Geneious Prime version 2020.1.1 (https://www.geneious.com) for coverage analysis before consensus calling, and consensus sequences were generated by using iVar version 1.2.2 (https://github.com/andersen-lab/ivar).

Genome sequences of SARS-CoV-2 from Western Australia were assigned to lineages (*7*) by using the Phylogenetic Assignment of Named Global Outbreak LINeages (PANGOLIN) tool (https://github.com/cov-lineages/pangolin). On July 17, 2020, we retrieved SARS-CoV-2 complete genomes with corresponding metadata from the GISAID database (https://www.gisaid.org/epiflu-applications/next-hcov-19-app). The final dataset contained 540 GISAID whole-genome sequences that were aligned with the sequences from Western Australia generated in this study by using MAFFT version 7.467 (https://mafft.cbrc.jp/alignment/software). Phylogenetic trees were visualized in iTOL (Interactive Tree Of Life, https://itol.embl.de) and MEGA version 7.014 (*8*). At the time of sequencing, the virologists and scientists overseeing and performing the WGS were blinded as to the epidemiologic details (Appendix).

Virus culture was attempted for primary samples sent directly to the reference laboratory but not for samples received after primary testing at another laboratory. Clinical specimens were inoculated in triplicate wells with Vero-E6 cells at 80% confluency, incubated at 37°C in 5% CO_2_, and inspected for cytopathic effect daily for up to 10 days. Identity was confirmed by in-house PCRs as described for previous sequences.

### Determining Likely Source of Infection

The likely source of infection for each passenger with PCR-confirmed SARS-CoV-2 infection was determined by using the results of WGS and epidemiologic investigations that assessed the potential for other sources of exposure during the incubation period. For this investigation, we considered primary cases to be passengers with SARS-CoV-2 infection identified by PCR in Western Australia who probably acquired their infection before boarding the flight. 

We defined primary cases as passengers with SARS-CoV-2 who had been on a cruise ship with a known outbreak in the 14 days before illness onset and whose specimen yielded a virus genomic sequence closely matching that of the ship’s outbreak strain; any passenger whose illness began before or within 48 hours after the flight’s departure (i.e., infectious during the flight); or both. We defined secondary cases as passengers with PCR-confirmed SARS-CoV-2 infection who had not been on a cruise ship with a known SARS-CoV-2 outbreak within 14 days of illness onset and in whom symptoms developed >48 hours after and within 14 days of the flight. We further characterized secondary cases as being flight associated if they were in international passengers who arrived in Sydney on March 19 or domestic Australia travelers who had not been on a cruise ship in the 14 days before illness and whose specimens yielded a WGS lineage not known to be in circulation at their place of origin but that closely matched the lineage of a primary case on the flight. Secondary cases among persons who had recently been on a cruise ship with no known onboard SARS-CoV-2 transmission or domestic passengers who had not been on a cruise ship but for whom WGS information was unavailable were categorized as possibly flight associated (Table 2). 

**Table 2:** Criteria for flight-associated and possibly flight-associated secondary cases of SARS-CoV-2 infection in persons aboard a flight from Sydney to Perth, Australia, March 19, 2020

Secondary case classification	Epidemiologic criteria	Virus WGS criteria
Flight associated	International passenger who arrived at Sydney International Airport on March 19, 2020, and transited to the flight OR domestic passenger in Australia not associated with a cruise ship before illness	Specimen yielded lineage not circulating in place of origin AND Lineage closely matched that of passengers with primary cases on flight
Possibly flight associated	Passenger on cruise ship with no known SARS-CoV-2 transmission identified	Specimen from passenger or traveling companion yielded lineage related to that of passengers with primary cases on flight
	Domestic passenger in Australia not associated with a cruise ship before illness	WGS data not available
*WGS, whole-genome sequencing.		

### Statistical Analyses

We tested the hypothesis that the risk for flight-associated infections was independent of seat assignment, as might occur if secondary infections were acquired while awaiting the flight in Sydney or after disembarking in Perth. We compared the proportion of secondary cases observed between aircraft cabins and between seating types (i.e., window vs. nonwindow seats) by calculating risk ratios with corrected Mantel-Haenszel χ^2^ tests and using EpiInfo Statcalc version 7.2.0.1 (https://www.cdc.gov/epiinfo).

## Results

**Figure 1:**
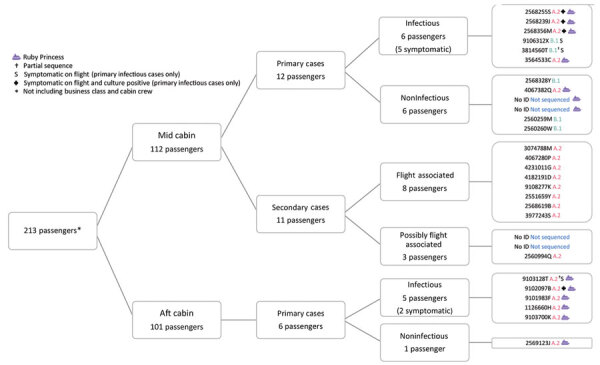
Distribution of SARS-CoV-2 infection cases among passengers on a flight from Sydney to Perth, Australia, on March 19, 2020. Far right column shows passenger identification numbers and SARS-CoV-2 lineage determined by whole-genome sequencing (A.2, B.1, not determined).

**Figure 2:**
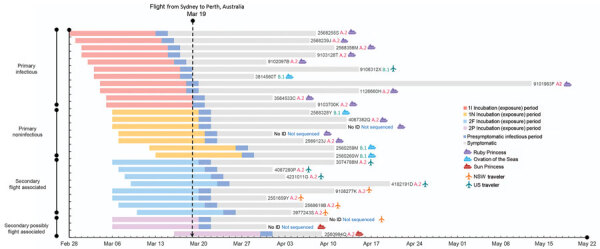
Theoretical maximum incubation (exposure), infectious period, and symptomatic period until time of resolution of illness in passengers on flight from Sydney to Perth, Australia, on March 19, 2020, with primary and secondary SARS-CoV-2 cases, by place of journey origin. Passenger identification numbers and SARS-CoV-2 lineage determined by whole-genome sequencing (A.2, B.1, not determined) indicated to right of bars. NSW, New South Wales, Australia; 1I, primary case, infectious; 1N, primary case, noninfectious; 2F, secondary case, flight associated; 2P, secondary case, possibly flight associated.

A total of 64 passengers on the flight had or later experienced an illness compatible with COVID-19 and were tested by PCR; 29 were SARS-CoV-2 positive (Figure 1) and 35 were negative. Among PCR-confirmed cases, symptom onset occurred during March 15–April 1, 2020 (Figure 2); median age was 59 (range 4–81) years, 14 were female, and 15 were male. Moreover, 13 had been passengers on the Ruby Princess, 4 on the Ovation of the Seas, and 2 on the Sun Princess. Five others were international travelers transiting through Sydney, and 5 were domestic travelers within Australia who had not been on a cruise ship.

**Figure 3:**
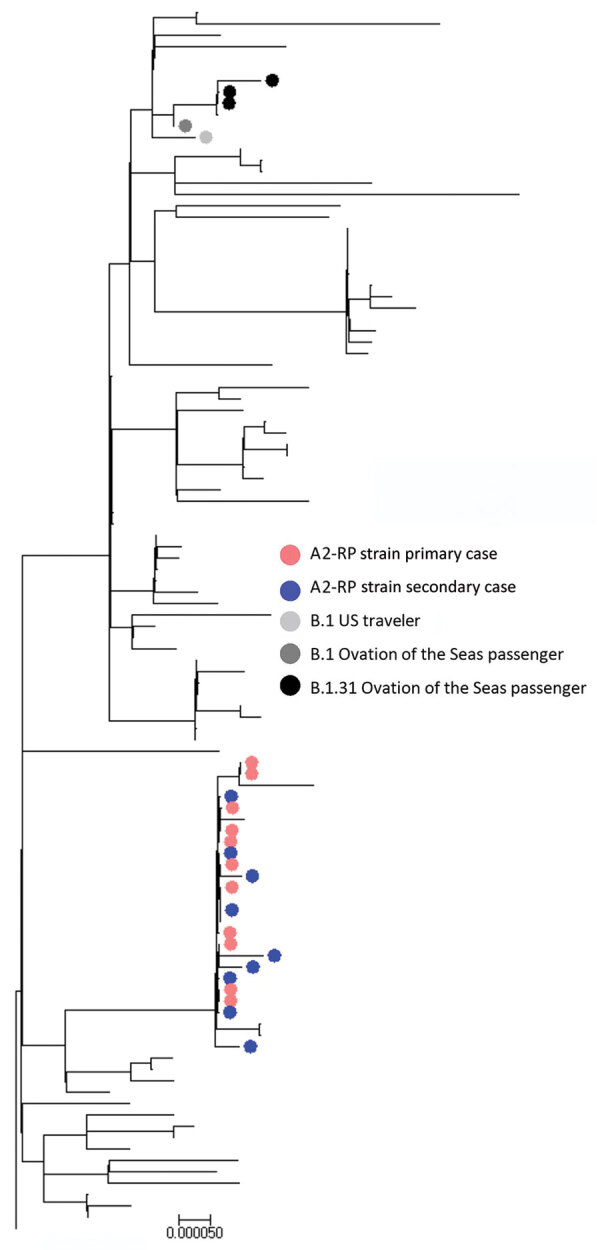
Phylogenetic tree generated in MEGA version 7.0.14 (*8*) for all SARS-CoV-2 whole-genome sequences with >80% genome coverage. Colors indicate samples from 458 persons with cases linked to cluster on flight from Sydney to Perth, Australia, on March 19, 2020. Scale bar indicates nucleotide substitutions per site.

Sufficient viral RNA was available to generate an adequate sequence for 25 of the 29 samples positive by PCR; 100% coverage was obtained for 21 and partial coverage (81%–99%) for 4 samples. The phylogenetic tree for the 21 complete genomes (Figure 3; Appendix Figure, panel A) showed that they belonged to either the A.2 (n = 17) or B.1 (n = 4) sublineages of SARS-CoV-2. All of the complete A.2 sequences belonged to a distinct genomic cluster separated by <2 single-nucleotide polymorphisms (Appendix Figure, panel B), which we designated as A2-Ruby Princess (A2-RP). The A2-RP strain had not been identified on the GISAID international database before this outbreak (*9*). The 4 B.1 viruses comprised 3 B.1.31 and 1 phylogenetically more distant B.1 strain. Of the 4 partial sequences, 3 clustered with the A2-RP strains and the other was designated B.1.1 and was phylogenetically close to the B.1.31 sequences (Figure 3). We attempted to culture 17 PCR-positive specimens, 9 of which grew SARS-CoV-2.

### Primary Cases

Of the 29 cases of PCR-confirmed SARS-CoV-2 linked to the flight, 18 were classified as primary cases. Former Ruby Princess passengers accounted for 13 of the primary cases, and all viruses with WGS available were A2-RP strain. Nine Ruby Princess passengers were classified as infectious during the flight based on illness onset; 4 specimens collected on the day after the flight (March 20) were culture positive. Of the remaining 5 primary cases, 4 were in passengers from the Ovation of the Seas (1 classified as infectious during the flight); SARS-CoV-2 virus was B.1.31 for 3 passengers, and for the other passenger a partial B.1.1 sequence clustered near the B.1.31 viruses. Virus of the fifth B.1 lineage was obtained from a traveler returning from the United States who was also infectious during the flight (Figure 3; Appendix Figure 1, panel B).

### Secondary Cases

We identified 11 secondary cases with symptom onset during March 22–April 1; among these, 8 cases were classified as flight associated. These 8 persons did not know each other. Four had commenced their journeys from different US cities and had taken an overnight flight from Los Angeles, California, USA, which landed at Sydney Airport on the morning of March 19. All had viruses of the A2-RP strain (3 by full and 1 by partial sequence; Figure 2), which was not circulating in the United States at the time of the flight. As of July 28, 2020, the GISAID database contained 5 sequences from 2 US sites that matched the A2-RP strain; relevant public health authorities confirmed that these sequences were obtained from passengers returning to the United States after disembarking from the Ruby Princess in Australia with specimen collection dates on or after March 22.

The remaining 4 flight-associated cases flew from various locations in New South Wales. Two resided in Sydney, 1 took a short flight from regional New South Wales, and 1 traveled by car several hours to Sydney Airport on March 19. None were previously identified contacts of a person with COVID-19 or had any known interactions with cruise ship passengers before the flight. All had virus with the A2-RP strain. There was no evidence of A2-RP strain circulation in Sydney before disembarkation of passengers from the Ruby Princess on March 19 (*9*,*10*). The median duration from day of flight to illness onset for the 8 persons with flight-associated infections was 4 (range 3–6) days.

Three secondary cases were classified as possibly flight associated. Two traveling companions disembarked from the Sun Princess cruise on March 19; there was no outbreak of SARS-CoV-2 on this cruise ship, and neither person had known contact with anyone with COVID-19. Virus was the A2-RP strain for 1 of these persons, but no specimen was available for WGS for the other. One case in a domestic passenger who spent the incubation period in regional New South Wales was classified as possibly rather than flight associated because no specimen was available for WGS.

Overall, the risk of acquiring SARS-CoV-2 in New South Wales or Western Australia from an unknown community source during this time was low. On the day of the flight, New South Wales (population 7.5 million) reported a cumulative total of 307 COVID-19 infections, of which only 26 (3.5 cases/1,000,000 population) were locally acquired from an unknown source (*11*); in Western Australia (population 2.6 million), 52 cumulative cases had been identified, of which only 2 were locally acquired from an unknown source (0.8 cases/1,000,000 population) (*12*).

### Spatial Distribution during Flight

**Figure 4:**
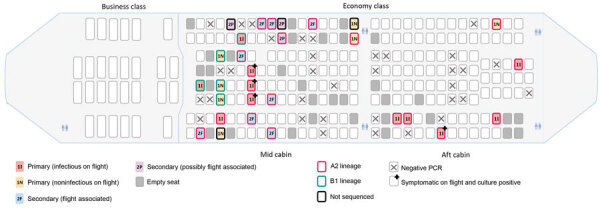
Spatial distribution of primary (infectious and noninfectious) and secondary (flight-associated and possibly flight-associated) cases of SARS-CoV-2 aboard flight from Sydney to Perth, Australia, on March 19, 2020. Passengers are identified by place of origin and SARS-CoV-2 lineage as determined by whole-genome sequencing. 1I, primary case, infectious; 1N, primary case, noninfectious; 2F, secondary case, flight associated; 2P, secondary case, possibly flight associated.

Eleven primary cases (6 passengers in mid cabin and 5 in aft cabin) were classified as having been infectious during the flight; 5 were symptomatic. The mid cabin had 3 symptomatic passengers from the Ruby Princess seated together in the same row in the middle section; all had A2-RP (full sequence) viruses and positive virus culture results (Figure 4). In the mid cabin were 1 infectious traveler from the United States, 1 from Ovation of the Seas (both with B.1 lineage virus and culture-negative results), and 1 additional Ruby Princess passenger who was presymptomatic but infectious. The remaining 5 infectious primary cases (all Ruby Princess passengers) were seated in the aft cabin; 2 were symptomatic during the flight. All secondary cases occurred in persons seated in the economy class mid cabin.

Among secondary cases, 8 passengers were seated within 2 rows of infectious Ruby Princess passengers and 3 were more distant (2 possibly flight-associated cases were seated 3 rows away and 1 flight-associated case was seated 6 rows away). Seven (64%) secondary cases were among persons who had window seats (Figure 4).

### Risk for Infection by Cabin and Seat Position

The risk for SARS-CoV-2 secondary infections among passengers seated in the mid cabin (11 cases/112 passengers) was significantly greater than for those seated in the aft cabin (0 cases/101 passengers; risk ratio undefined; corrected Mantel–Haenszel χ^2^ = 8.6; p<0.005). The secondary attack rate among mid-cabin passengers in window seats (7 cases/28 passengers) was significantly greater than among those not in window seats (4/83; risk ratio 5.2; 95% CI 1.6–16.4; corrected Mantel–Haenszel χ^2^ = 7.0; p<0.007).

## Discussion

This combination of comprehensive epidemiologic investigation and WGS analysis builds a strong case for flight-associated transmission of SARS-CoV-2. Convergence of the following 3 factors enabled this investigation: 1) the emergence of a unique SARS-CoV-2 strain (A2-RP) among cruise ship passengers disembarking in Sydney at the time of the flight, 2) identification of the A2-RP virus sequence among travelers arriving from overseas who did not leave the Sydney Airport before transiting to the flight, and 3) the very limited community transmission of SARS-CoV-2 within Australia at the time.

Other published reports describe suspected flight-associated transmission of SARS-CoV-2 (*2*,*3*,*13*,*14*), but these reports lack supportive genomic evidence. Our investigation demonstrates the value of WGS for elucidating transmission of SARS-CoV-2. Without genomic evidence, we would have assumed that the overseas travelers on this flight acquired their infection in the United States rather than within Australia.

We report several other findings. First, 3 of the 11 persons with secondary infections were outside the usual parameters used to identify close contacts of an infectious passenger on an airplane (2 rows in front and behind). On the flight described, passengers with flight-associated infection spanned 9 rows and were on opposite sides of the airplane. Discussion with airline representatives indicated that no air handling maintenance issues were logged for this flight and no illnesses were reported among the crew (Director of Medical Services for the airline, pers. comm., 2020 Sep 8). Although the results of 1 study of SARS-CoV-1 indicated a significantly increased risk for infection among passengers seated within 2 or 3 rows in front of an index case (*15*), secondary cases have also been reported for passengers seated farther away (*16*). A report funded by the US Federal Aviation Administration concluded that human movement might explain the transmission of SARS-CoV-1 “to passengers seated as far as seven rows from the infected passenger during the SARS outbreak in 2003” (*17*). The spatial distribution of secondary cases on this flight suggests that the current public health practice for contact tracing of passengers exposed to SARS-CoV-2 while aboard aircraft may benefit from additional study (*18*,*19*).

Second, most of the secondary infections were in persons seated at the window, 2 of whom denied ever leaving their seat during the flight. This finding was unanticipated given the widely held view that persons in window seats are at lower risk for exposure to an infectious pathogen during flight, a belief supported by data simulating transmission of droplet-mediated respiratory illnesses during flights of similar duration on single-aisle airplanes in the United States (*20*). Other studies, however, have emphasized the difficulty of measuring and understanding the complex airflow inside an aircraft cabin, even under steady-state conditions (*17*). In addition, movement of passengers and crew can also affect airflow patterns on board; further study of the dynamics of airflow on aircraft under real-world conditions is warranted.

Third, the risk for secondary SARS-CoV-2 infections from an infectious passenger during flight does not seem to be uniform. On this flight, there were 2 potentially infectious persons with a B lineage virus, but no B lineage secondary infections were identified. Furthermore, several persons with potentially infectious primary cases with A2-RP virus strain were in the mid and aft cabins (4 mid cabin and 5 aft cabin), and yet no secondary cases were identified in the aft cabin. This disparity raises the possibility that there was >1 SARS-CoV-2 superspreader in the mid cabin during the flight (*21*). Although no reports of unwell passengers were logged with the airline for this flight (Director of Medical Services for the airline, pers. comm., 2020 Sep 8), 5 of the 8 passengers with flight-associated secondary cases reported having noted coughing passengers. Anecdotal information obtained via interviews indicates that mask use was rare among the passengers overall, including those who had respiratory symptoms. Of note, 2 passengers with secondary cases reportedly wore masks during the flight but not for the entire flight. Although semiquantitative data on comparative viral loads based on cycle threshold values was available, we could not use these data to further investigate the role of upper respiratory tract viral load for determining transmission risk because samples were collected after the flight. Therefore, viral loads of passengers during the flight are unknown. Of note, 4 persons who were infectious on the flight had culture-positive specimens collected the next day (March 20; Figure 4).

Reports of suspected in-flight transmission of SARS-CoV-2 are relatively few, which probably reflects the challenges of establishing in-flight transmission; the fact that flight-associated transmission may be rare; and the fact that as the pandemic progressed, many airlines adopted measures to decrease risk (e.g., reduced food and beverage services, removal of in-flight entertainment, provision of masks and sanitizing wipes, limitation of movement around the cabin, and enhanced cleaning of the airplane) (*22*). The Australian Health Protection Principal Committee has endorsed a Domestic Passenger Journey Protocol to provide clear guidance regarding risk-minimization principles and processes in domestic airports and on airplanes for domestic passengers (*23*,*24*). This guidance also entails “reminding people not to travel if unwell” (*23*,*24*). As awareness of the threat posed by COVID-19 on cruise ships has grown, it is unlikely that the circumstances that led to exposures on this flight will be repeated.

This study has several limitations. First, we cannot exclude the possibility that the 3 passengers with possibly flight-associated infection might have been exposed before or after their journey; however, the very low levels of community transmission in Australia at the time, combined with the known proximity of these travelers to infectious persons on the airplane, suggest that this possibility is unlikely. Conversely, we cannot rule out the possibility that the 7 passengers with primary cases who had disembarked from a cruise ship with known SARS-CoV-2 illnesses on board but in whom symptoms developed >48 hours after the flight acquired their infection aboard the airplane; however, given the balance of probabilities, it is more likely that they acquired their infections on the ship. Second, lack of detailed information for all passenger movements within the airport, at the boarding gate, and aboard the aircraft also limits our ability to determine with specificity where flight-associated exposures occurred. However, the spatial clustering of all secondary cases within the mid cabin suggests that transmission most likely occurred aboard the airplane. If persons had been exposed principally at the airport, one might expect the 11 secondary cases to have been distributed beyond the mid cabin. On this flight, all passengers were invited to board at the same time, rather than by rows. Third, categorizing cases as primary or secondary was predicated on the passenger’s self-reported date of symptom onset, which may have been subject to recall errors. Fourth, case ascertainment bias is possible because although all passengers were informed of their potential exposure, passengers who had disembarked from a cruise ship that subsequently had a widely publicized outbreak or the close contacts of a primary case who were actively monitored for 14 days may have been more likely to seek testing if they became ill. Last, we cannot be certain that we captured all SARS-CoV-2 infections among persons who traveled on this flight because PCR testing was limited to passengers who reported symptoms and testing is not 100% sensitive (*25*). Given that flight-associated transmission of SARS-CoV-2 from asymptomatic persons has been reported, it is possible that some persons with secondary infections were exposed to passengers other than the infectious cases we identified on the flight (*14*); thus, our findings on the spatial distribution of primary and secondary cases should be interpreted with caution. Attempts to identify additional primary and secondary cases by using SARS-CoV-2 serologic testing are ongoing.

In conclusion, this study documents transmission of SARS-CoV-2 associated with a medium-duration domestic flight within Australia. It also demonstrates the value of WGS for determining SARS-CoV-2 transmission. 

Appendix Supplemental methods and results from study of flight-associated transmission of severe acute respiratory syndrome coronavirus 2 corroborated by whole-genome sequencing.
