# Supplementary material for: Flight-Associated Transmission of Severe Acute Respiratory Syndrome Coronavirus 2 Corroborated by Whole-Genome Sequencing
Source: Emerg Infect Dis. 2020 Dec;26(12):2872–80. doi: 10.3201/eid2612.203910 (PMC7706937; doi:10.3201/eid2612.203910)
Supplement: Appendix — Supplemental methods and results from study of flight-associated transmission of severe acute respiratory syndrome coronavirus 2 corroborated by whole-genome sequencing. [file 20-3910-Techapp-s1.pdf]

Article DOI: https://doi.org/10.3201/eid2612.203910

Flight-Associated Transmission of Severe Acute Respiratory Syndrome Coronavirus 2 Corroborated by Whole-Genome Sequencing

Appendix

Detailed methods

Samples

Clinical samples included in this study were collected between February 22 and April 04 2020. Nasopharyngeal secretions were collected from each nostril of individuals suspected of SARS-CoV-2 infection using a flocked nasopharyngeal (NP) swab (FLOQ Swabs™, Copan Diagnostics, USA). The swab was placed in 1ml of virus transport media (VTM) and transported at 4-8°C prior to testing.

Nucleic acid extraction

Nucleic acid was extracted from 200µL of clinical sample by a semi-automated MagMAX-96 magnetic bead-based purification system (ThermoFisher, Australia). In house modifications to the manufacturer's protocol were made as cited by Chidlow et al. (*1*). An automated liquid-handling robot (Hamilton,MA,USA) was used to add nucleic acid extract into the SARS-CoV-2 PCR master mix.

PCR Diagnostic assays performed at Pathwest Laboratory Medicine WA

Two sets of PCR assays were used to detect SARS-CoV-2 RNA from clinical samples. The first set was designed and validated in-house and the second set was the envelope gene PCR described by Corman et al. (*2*). The in-house validated one step real-time reverse transcription PCR (RT-PCR) comprised primers and probe directed to a 63 bp region of the SARS-CoV-2 spike (S) gene. The nucleotide position of the forward primer, reverse primer and hydrolysis probe (supplemental table 1) were based on the full genome of the reference SARS-CoV genome

(NCBI Genbank accession MN908947). The 20µl one step RT-qPCR mix was prepared with 1x Quanta qScript XLT One-Step RT-qPCR Tough Mix (Quantabio, USA). Assay performance parameters including analytical (and clinical) sensitivity, specificity, repeatability and reproducibility were evaluated following the Minimum Information for Publication of Quantitative Real-Time PCR Experiments (Bustin et al. (*3*) and to the requirements of the National Pathology Accreditation Advisory Council (NPAAC) (*4*).

Thermocycling conditions were as follows 10 min at 50˚C, 1 min incubation at 95˚C then 40 cycles of 20s at 95˚C and 80s at 60˚C using the CFX96 Touch Real-Time PCR Detection System (BIO-RAD, Australia). All tests comprised duplicate positive control samples as well as a non-template control sample interspersed every 5 samples. Fluorescence data was automatically collected in the annealing–extension phase of the of each PCR cycle.

Tiled amplicon PCR and whole genome sequencing

Previously positive samples with a C_t value of less than 30 on the envelope gene PCR (*2*) were extracted by MagMax (*1*) and total nucleic acid was eluted in 50µL. Complementary DNA (cDNA) was synthesized from each nucleic acid extract using the SuperScript™ IV VILO™ (SSIV VILO) Master Mix System (ThermoFisher, Australia) and was used as input for the tiled amplicon PCR. The tiled amplicon PCR was performed as previously described (*5*). Briefly, sample cDNA was added to 14 PCR's, adhering to the primer scheme described in Table S2 and performed using the Platinum SuperFi II green master mix (ThermoFisher, Australia). The expected 2.5kb amplicon product from each PCR was size verified on a 1% gel and then pooled. Pooled samples were then purified using Ampure XP beads (Beckman Coulter, CA, USA) and then quantified using the Qubit 2.0 instrument prior to library preparation. Genomic libraries were prepared using Nextera XT DNA library preparation kit (Illumina, CA, USA), size distribution of the multiplexed library was performed by the Australian Genomic Research Facility (AGRF, Perth, Australia). The pooled library was sequenced using the MiSeq Micro kit, v2 reagent (300 cycles) on an Illumina MiSeq Instrument (Illumina, USA).

SARS-CoV-2 Genome Assembly

Base calling and demultiplexing of reads was performed automatically on the Illumina MiSeq instrument and sequencing quality metrics were assessed using fastQC v0.11.1 with only high quality libraries used in the downstream analyses. Adaptor and quality trimming were

performed using BBDuk (v38.84). Processed reads were mapped to the SARS-CoV reference genome (MN908947) using BBMap with default parameters then sorted using SAMtools. Primer sequences were trimmed from the sorted read mapped alignment file using iVar v1.2.1. Primer sequences were identified by comparing mapped position of the input alignment file to reference positon (MN908947) of primer sequences specified in a BED file. Primer-clipped alignment files were imported into Geneious Prime (v2020.1.1) for coverage analysis prior to consensus calling. Consensus sequences were generated using iVar with parameters set at 10 for minimum depth, 20 for minimum quality, and 0.1 for minimum frequency

SARS-CoV-2 Lineage assignment

Western Australian genome sequences of SARS-CoV-2 were assigned to lineages according to the nomenclature proposed by A. Rambaut et al. (*6*) using the Phylogenetic Assignment of Named Global Outbreak LINeages (PANGOLIN) tool (https://github.com/cov-lineages/pangolin).

Phylogenetic analysis

SARS-CoV A.2 complete genomes with corresponding metadata were retrieved from GISAID as of July 17 2020. GISAID sequences were only included for further analysis if they were >28000bp in length and contained <0.05% of ambiguous bases. The final dataset were aligned using MAFFT v7.467. The alignment was visually inspected using Geneious prime prior to phylogenetic analysis. Maximum Likelihood phylogenetic trees were inferred using IQTREE v2.06 incorporating a GTR+F+R3 nucleotide substitution model. Robustness of each node was assessed using UltraFast bootstrap approximation with 1000 replicates and an approximate likelihood-ratio test based on a Shimodaira-Hasegawa like procedure (SH-aLRT) with 1000 replicates.

List of GISAID accession numbers

The new sequences have been deposited in GISAID with accession IDs EPI_ISL_512712 - EPI_ISL_512765, EPI_ISL_470832 to EPI_ISL_470875 and EPI_ISL_420532 to EPI_ISL_420531.

References

1. Chidlow G, Harnett G, Williams S, Levy A, Speers D, Smith DW. Duplex real-time reverse transcriptase PCR assays for rapid detection and identification of pandemic (H1N1) 2009 and seasonal influenza A/H1, A/H3, and B viruses. J Clin Microbiol. 2010;48:862–6. 10.1128/jcm.01435-09 PubMed https://doi.org/10.1128/JCM.01435-09

2. Corman VM, Landt O, Kaiser M, Molenkamp R, Meijer A, Chu DK, et al. Detection of 2019 novel coronavirus (2019-nCoV) by real-time RT-PCR. Euro Surveill. 2020;25:2000045. 10.2807/1560-7917.ES.2020.25.3.2000045 PubMed https://doi.org/10.2807/1560-7917.ES.2020.25.3.2000045

3. Bustin SA, Benes V, Garson JA, Hellemans J, Huggett J, Kubista M, et al. The MIQE guidelines: minimum information for publication of quantitative real-time PCR experiments. Clin Chem. 2009;55:611–22. 10.1373/clinchem.2008.112797 PubMed https://doi.org/10.1373/clinchem.2008.112797

4. National Pathology Accreditation Advisory Council. Regulatory requirements for in-house IVDs. Australian Capital Territory: Therapeutic Goods Administration; 2018.

5. Eden J-S, Rockett R, Carter I, Rahman H, de Ligt J, Hadfield J, et al. An emergent clade of SARS-CoV-2 linked to returned travellers from Iran. Virus Evolution. 2020;6: veaa027. https://doi.org/10.1093/ve/veaa027

6. Rambaut A, Holmes EC, Hill V, O'Toole Á, McCrone J, Ruis C, et al. A dynamic nomenclature proposal for SARS-CoV-2 to assist genomic epidemiology. Nat Microbiol. 2020. https://doi.org/10.1101/2020.04.17.046086

Appendix Table. Sequences of primers used for amplicon generation

PRIMER	SEQUENCE (5' - 3')	Length (nt)	Tm (ºC)	Start (nt)#	End (nt)#	Length (nt)	Orientation
nCoV_2019_1L	ACCAACCAACTTTCGATCTCTTGT	24	60.7	31	54	2562	Forward
nCoV_2019_8R	GCTTCAACAGCTTCACTAGTAGGT	24	60.6	2,569	2,592		Reverse
nCoV_2019_15L	ACAGTGCTTAAAAAGTGTAAAAGTGCC	27	61.3	4,295	4,321	2579	Forward
nCoV_2019_22R	ACAGTATTCTTTGCTATAGTAGTCGGC	27	60.7	6,847	6,873		Reverse
nCoV_2019_29L	ACTTGTGTTCCTTTTTGTTGCTGC	24	61.4	8,596	8,619	2479	Forward
nCoV_2019_36R	GAACAAAGACCATTGAGTACTCTGGA	26	60.7	11,049	11,074		Reverse
nCoV_2019_43L	TACGACAGATGTCTTGTGCTGC	22	60.9	12,711	12,732	2536	Forward
nCoV_2019_50R	TAACATGTTGTGCCAACCACCA	22	61	15,225	15,246		Reverse
nCoV_2019_57L2	ACTATGGTGATGCTGTTGTTTACCG	25	61.6	16,847	16,871	2432	Forward
nCoV_2019_64R2	ACCAGGCAAGTTAAGGTTAGATAGC	25	60.6	19,254	19,278		Reverse
nCoV_2019_71L	ACAAATCCAATTCAGTTGTCTTCCTATTC	29	60.5	21,358	21,386	2490	Forward
nCoV_2019_78R	TGTGTACAAAAACTGCCATATTGCA	25	60.2	23,823	23,847		Reverse
nCoV_2019_85L	ACTAGCACTCTCCAAGGGTGTT	22	61	25,602	25,623	2571	Forward
nCoV_2019_92R	AGGTTCCTGGCAATTAATTGTAAAAGG	27	60.5	28,146	28,172		Reverse
nCoV_2019_7L	ATCAGAGGCTGCTCGTGTTGTA	22	61.7	1876	1897	2575	Forward
nCoV_2019_14R	AGTTTCCACACAGACAGGCATT	22	60.4	4,429	4,450		Reverse
nCoV_2019_21L2	TGGTGTATACGTTGTCTTTGGAGC	24	61.1	6287	6310	2565	Forward
nCoV_2019_28R2	CACTTCTCTTGTTATGACTGCAGC	24	60.1	8,828	8,851		Reverse
nCoV_2019_35L	TGTTCGCATTCAACCAGGACAG	22	61.4	10,363	10,384	2440	Forward
nCoV_2019_42R	CCTACCTCCCTTTGTTGTGTTGT	23	60.7	12,780	12,802		Reverse
nCoV_2019_49L	AGGAATTACTTGTGTATGCTGCTGA	25	60.6	14,546	14,570	2607	Forward
nCoV_2019_56R	ACACTATGCGAGCAGAAGGGTA	22	61.2	17,131	17,152		Reverse
nCoV_2019_63L	TGTTAAGCGTGTTGACTGGACT	22	60.2	18,897	18,918	2559	Forward
nCoV_2019_70R	TGACCTTCTTTTAAAGACATAACAGCAG	28	60.3	21,428	21,455		Reverse
nCoV_2019_77L	CCAGCAACTGTTTGTGGACCTA	22	60.7	23,123	23,144	2551	Forward
nCoV_2019_84R	AGGTGTGAGTAAACTGTTACAAACAAC	27	60.4	25,647	25,673		Reverse
nCoV_2019_91L	TCACTACCAAGAGTGTGTTAGAGGT	25	60.9	27,447	27,471	2420	Forward
nCoV_2019_98R	TTCTCCTAAGAAGCTATTAAAATCACATGG	30	60	29,837	29,866		Reverse

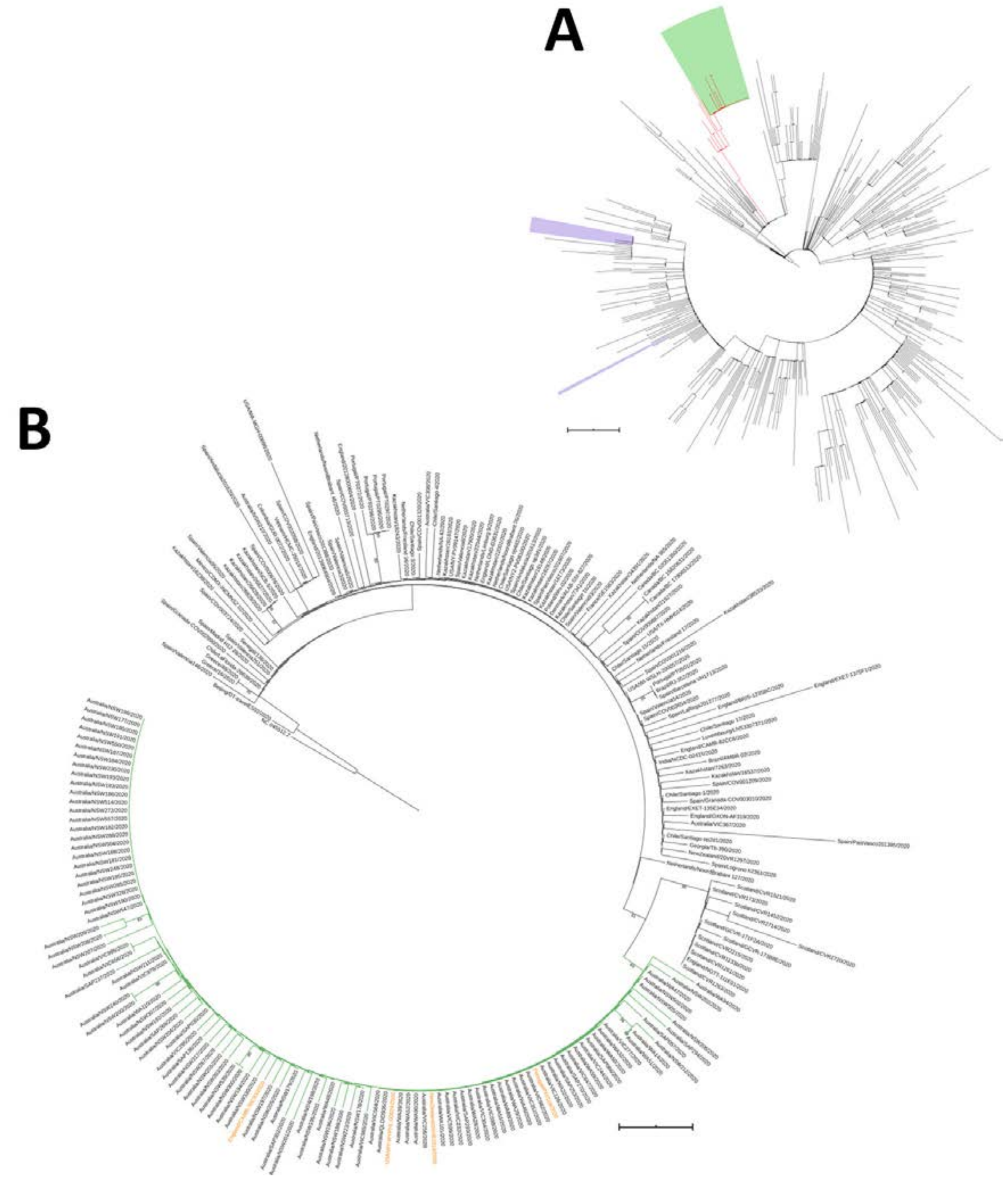

Appendix Figure. Maximum likelihood phylogenies of SARS-CoV-2 full genome sequences in the GISAID database as of July 17 2020. Sequences were aligned using MAFFT, partial sequences excluded in Geneious, maximum likelihood trees constructed using IQTree with 1000 bootstrap replicates and visualised on iToL. Tree scales correspond to 0.0001 substitutions. A) Sequences obtained from GISAID and selected to represent all lineages, including sequences from this cluster. Lineage A.2 is colored red, and the colored ranges represent the A2-RP strain (green) and the B.1 flight-associated cases (lilac). B) Global phylogeny of the A.2 lineage. The links within the A.2.RP clade are colored green and the sequences reported from outside Australia are in orange.
